# Supplementary material for: Terpene Profiles Composition and Micromorphological Analysis on Two Wild Populations of Helichrysum spp. from the Tuscan Archipelago (Central Italy)
Source: Plants (Basel). 2022 Jun 29;11(13):1719. doi: 10.3390/plants11131719 (PMC9269274; doi:10.3390/plants11131719)
Supplement: Supplementary file 1 [file plants-11-01719-s001.zip › plants-1717315-supplementary.pdf]

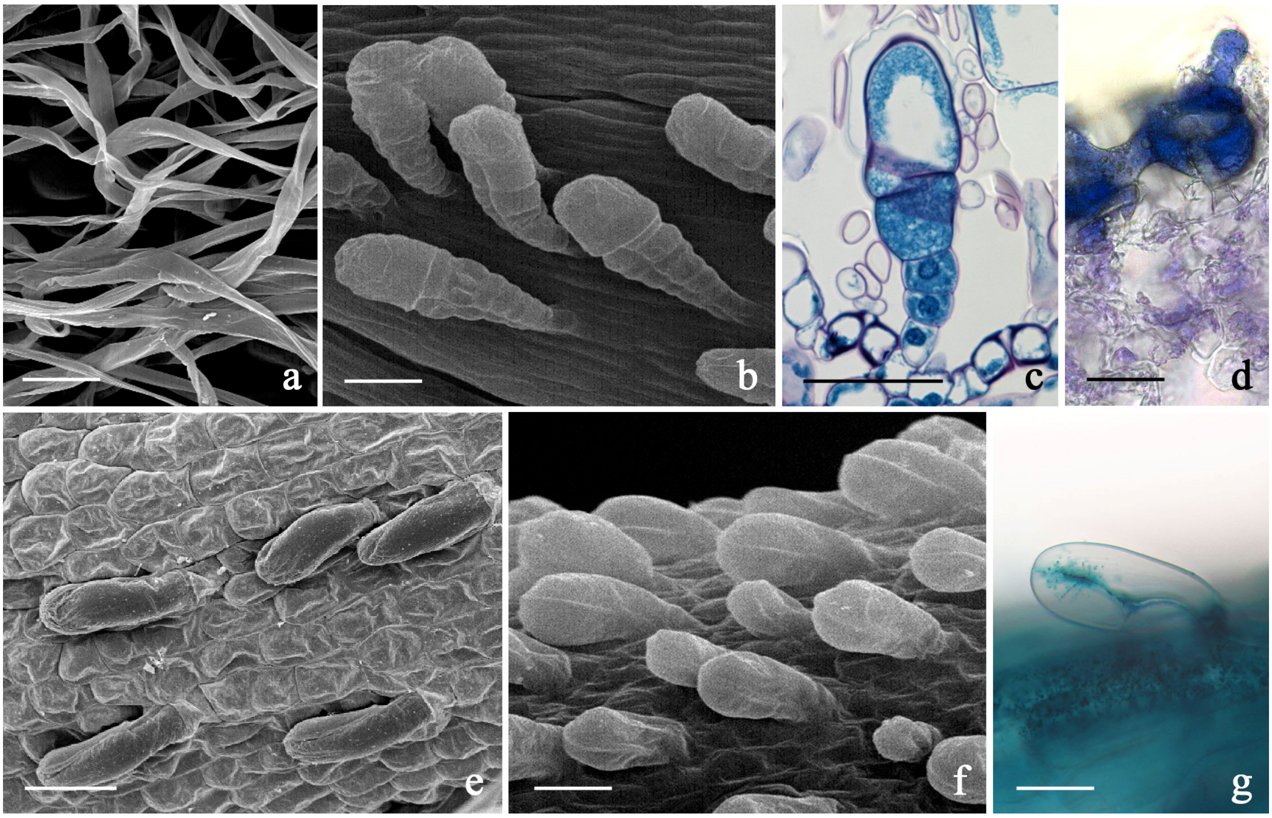

Figure S1. a-f. Trichomes morphotypes in the investigated *Helichrysum* populations. **a.** SEM micrograph showing the long filamentous non-glandular hairs. **b-d.** Micrographs showing the club-shaped glandular trichome morphotypes: general view (b, SEM); longitudinal section stained with Toluidine Blue (c, LM); terpenoidic nature of the secreted material stained with Nadi reagent (d, LM). **e-g.** Micrographs showing the duplex glandular trichome morphotypes: general views of the ovary surface in population A samples (e, SEM) and population B samples (f, SEM); muco-polisaccharidic nature of the secreted material stained with Alcian (g, LM). *Scale bars = 25 µm.*

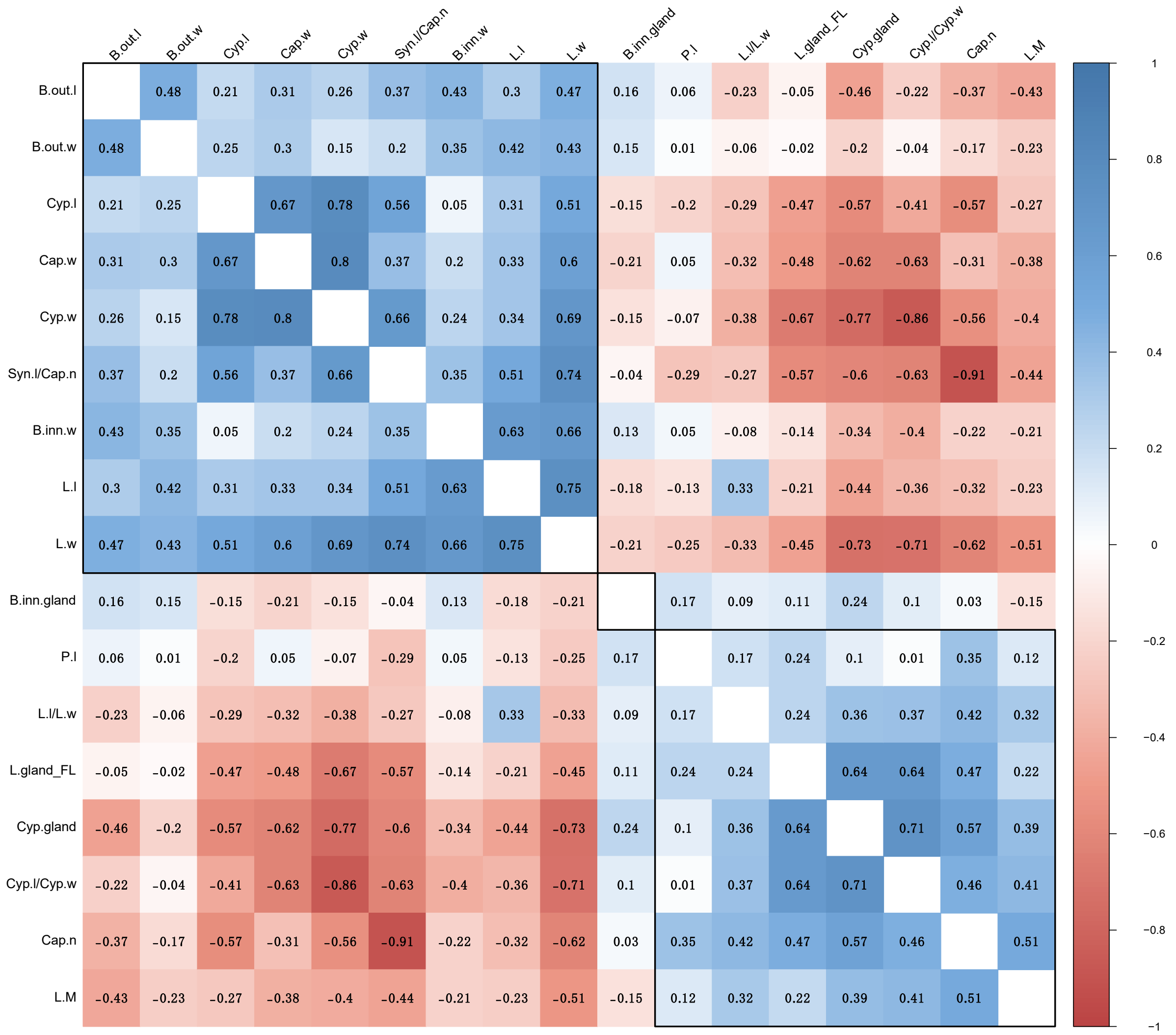

Figure S2. Spearman's correlation coefficients among morphological significant characters.

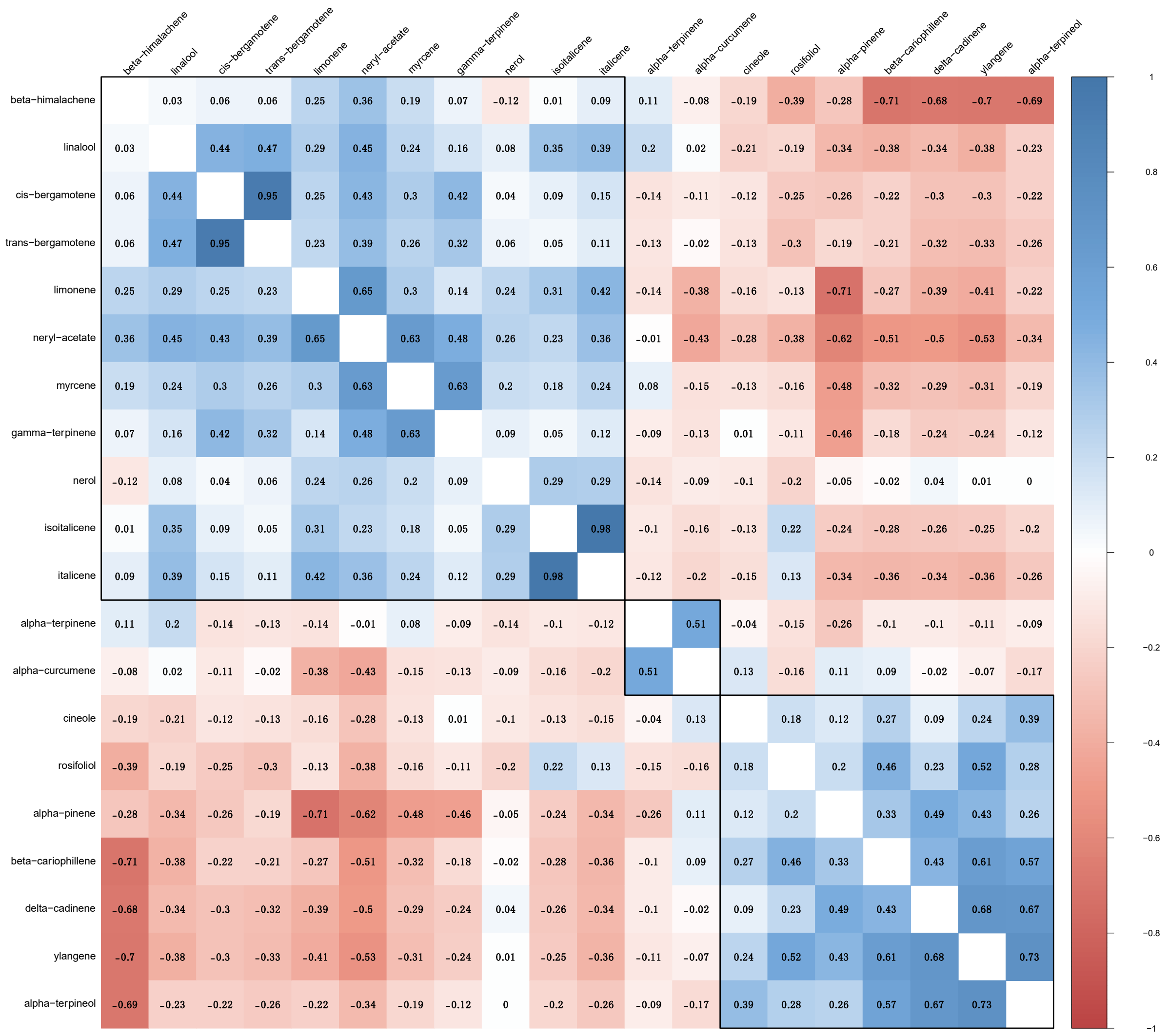

Figure S3. Pearson's correlation coefficients among phytochemical significant compounds of flower tissue.

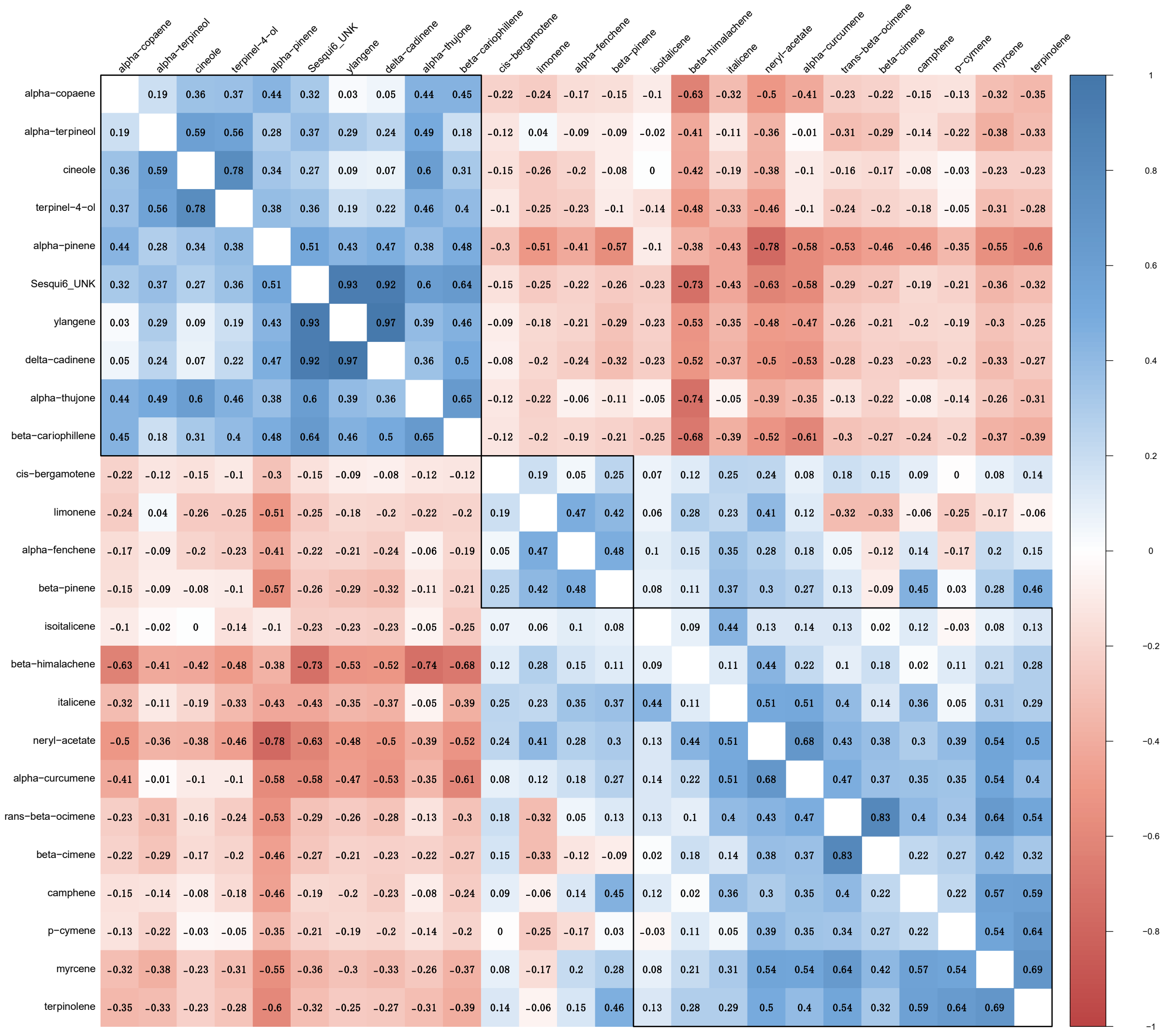

Figure S4. Pearson's correlation coefficients among phytochemical significant compounds of leaves tissue

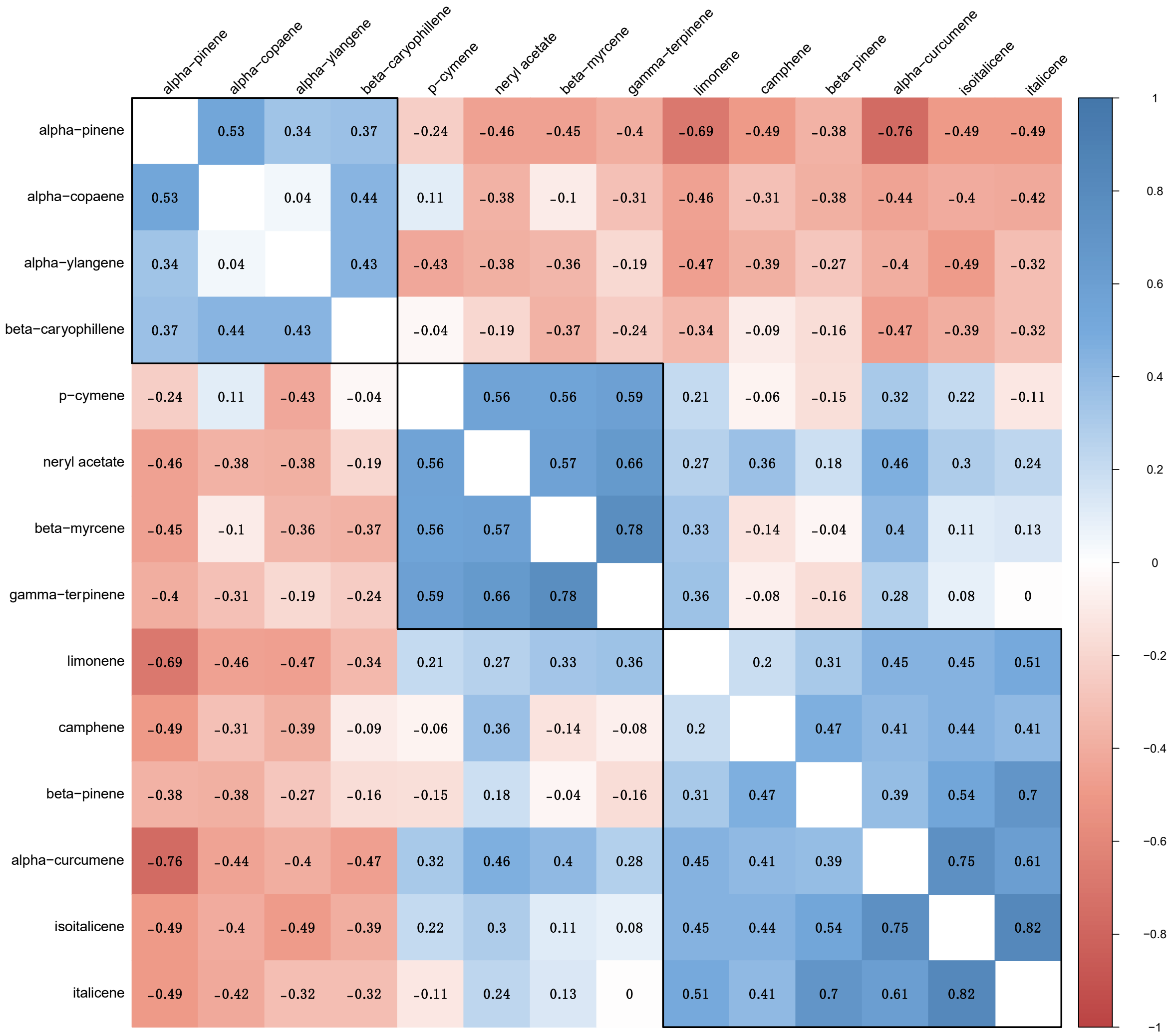

Figure S5. Pearson's correlation coefficients among phytochemical significant compounds of bark tissue.

Table S1. Contribution of morphological characters on the principal components (Dim.). The values reported are the square coordinates of the variables representing the contribution of the variables on the principal components. A high value indicates a good representation of the variable on the principal component.

	Dim.1	Dim.2	Dim.3	Dim.4	Dim.5
L.l	0.40	0.00	0.16	0.10	0.04
L.w	0.78	0.00	0.05	0.02	0.02
Cap.n	0.47	0.03	0.05	0.24	0.01
Cap.w	0.55	0.01	0.13	0.01	0.04
B.out.l	0.05	0.66	0.11	0.00	0.05
B.out.w	0.05	0.55	0.00	0.00	0.21
B.inn.w	0.04	0.09	0.33	0.35	0.05
B.inn.gland	0.09	0.31	0.06	0.00	0.28
P.l	0.08	0.19	0.30	0.13	0.14
Cyp.gland	0.72	0.00	0.02	0.02	0.00
Cyp.l	0.57	0.00	0.08	0.02	0.00
Cyp.w	0.77	0.01	0.05	0.00	0.05
L.gland_FL	0.37	0.11	0.01	0.03	0.01
L.M	0.23	0.03	0.06	0.28	0.16
Proportion of variance	36.96	14.12	99.70	86.49	7.63
Cumulative proportion	36.96	51.09	61.06	69.71	70.47

Table S2. Phytochemical analysis of flower, leaves and bark tissue from Capo d'Enfola (A) and Monte Capanne (B) population in Elba Island. Data are the mean of individuals ± SD. nd = not detected, tr: trace (< 0.1%). *H. litoreum* is reported in population A, and *H. italicum* subsp. *italicum* in population B. [a] MH = Monoterpene Hydrocarbon; OM = Oxygenated Monoterpene;; SH = Sesquiterpene Hydrocarbons; OS = Oxygenated Sesquiterpene. [b] Kovant's Retention Index. [c] Identification: "RI; MS" means that the compound was tentatively identified by Kovant's Retention Index (RI) and matches with database spectra in the NIST11 mass spectral library (MS); "Std" means that the identification of the molecules was confirmed by comparison of RI and the mass spectra with those of available standard compounds.

N	Compounds	Class of Compounds[a]	RT	RI[b]	Flower		Leaves		Bark		ID[c]
					A	B	A	B	A	B	
[1]	2-bornene	MH	9.17	985	0.21 ± 0.16	0.24 ± 0.29	nd	nd	nd	nd	RI;MS
[2]	α-pinene	MH	10.97	1030	58.64 ± 22.03	22.12 ± 18.93	55.1 ± 18.45	14.62 ± 7.74	53.41 ± 20.38	15.53 ± 15.02	Std
[3]	α-fenchene	MH	12.09	1072	2.17 ± 8.25	1.6 ± 1.91	3.41 ± 1.65	5.42 ± 3.33	15.83 ± 18.19	6.67 ± 7.3	Std
[4]	camphene	MH	12.15	1081	11.3 ± 16	4.08 ± 3.17	1.4 ± 1.48	2.68 ± 2.32	3.37 ± 2.49	19.3 ± 16.36	Std
[5]	β-pinene	MH	13.47	1124	2.14 ± 1.92	3.32 ± 2.82	2.86 ± 2.01	4.59 ± 1.72	0.56 ± 0.6	1.86 ± 1.72	Std
[6]	myrcene	MH	14.72	1172	0.31 ± 0.4	2.19 ± 2.41	0.56 ± 0.88	2.64 ± 1.95	1.36 ± 1.43	2.87 ± 2.77	Std
[7]	α-phellandrene	MH	15.31	1179	tr	tr	0.04 ± 0.04	0.1 ± 0.2	nd	nd	Std
[8]	α-terpinene	MH	15.75	1195	5.16 ± 13.4	3.51 ± 10.01	1.87 ± 3.91	1.38 ± 1.19	tr	tr	Std
[9]	limonene	MH	16.00	1215	18.27 ± 12.11	51.37 ± 16.24	30.85 ± 14.34	47.76 ± 21.38	22.11 ± 15.08	48.79 ± 18.41	Std
[10]	1,8-cineole	OM	16.66	1227	1.42 ± 2.96	0.12 ± 0.57	1.91 ± 2.85	0.19 ± 0.27	tr	tr	Std
[11]	(E)-β-ocimene	MH	17.11	1250	nd	nd	0.29 ± 0.59	2.78 ± 7.9	nd	nd	RI;MS
[12]	(Z)-β-ocimene	MH	17.66	1251	nd	nd	0.35 ± 0.76	11.27 ± 16.79	nd	nd	Std
[13]	γ-terpinene	MH	18.00	1258	1.75 ± 2.05	11.52 ± 16.14	1.37 ± 1.69	2.3 ± 5.18	0.52 ± 0.6	1.21 ± 1.08	Std
[14]	p-cymene	MH	18.46	1260	tr	tr	1.07 ± 1.55	5.74 ± 9.65	2.81 ± 3.92	3.74 ± 2.38	Std
[15]	terpinolene	MH	19.90	1298	tr	tr	0.76 ± 0.51	1.58 ± 0.88	nd	nd	Std
[16]	α-ylangene	SH	24.27	1507	9.69 ± 9.35	tr	3.47 ± 4.33	0.02 ± 0.1	4.56 ± 5.3	tr	RI;MS
[17]	α-copaene	SH	24.50	1515	0.09 ± 0.35	tr	16.08 ± 19.22	0 ± 0.01	12.54 ± 13.57	0.09 ± 0.3	RI;MS
[18]	α-thujone	OM	24.70	1519	nd	nd	1.39 ± 1.48	0.3 ± 0.57	nd	nd	Std
[19]	isoitalicene	SH	25.00	1521	0.2 ± 0.18	2.05 ± 2.9	0.94 ± 2.88	1.45 ± 1.36	3.84 ± 6.89	7.74 ± 3.12	RI;MS
[20]	linalool	OM	25.55	1546	0.82 ± 2.02	3.66 ± 2.97	0.77 ± 1.86	0.83 ± 0.78	nd	nd	Std
[21]	italicene	SH	25.98	1573	0.94 ± 0.83	7.21 ± 7.27	1.63 ± 2.06	5.44 ± 4.7	1.09 ± 0.87	3.85 ± 1.83	RI;MS
[22]	(Z)-α-bergamotene	SH	26.10	1580	0.36 ± 0.88	3 ± 3.94	0.34 ± 0.71	1.18 ± 1.76	nd	nd	RI;MS
[23]	(E)-α-bergamotene	SH	26.50	1591	0.51 ± 0.94	1.97 ± 2.5	nd	nd	nd	nd	RI;MS
[24]	terpinen-4-ol	OM	27.33	1616	0.49 ± 0.53	0.65 ± 0.89	0.37 ± 0.5	tr	nd	nd	Std
[25]	β-cariophillene	SH	27.45	1627	18.16 ± 17.64	1.35 ± 3.37	13.52 ± 13.46	1.21 ± 3.96	8.12 ± 12.06	2.62 ± 9.01	Std
[26]	β-himachalene	SH	29.30	1663	22.32 ± 16.18	34.33 ± 10.58	16.34 ± 15.54	29.23 ± 6.58	13.81 ± 10.95	15.71 ± 7.81	RI;MS
[27]	α-terpineol	OM	29.68	1684	0.58 ± 0.98	tr	0.93 ± 0.7	0.54 ± 0.28	nd	nd	Std
[28]	Sesqui6_UNK	SH	30.00	1692	nd	nd	6.2 ± 5.31	tr	nd	nd	RI;MS
[29]	neryl-acetate	OM	30.20	1730	1.86 ± 3.77	42.82 ± 15.48	2.3 ± 4.73	33.95 ± 8.05	2.42 ± 7.46	17.25 ± 13.71	RI;MS
[30]	δ-cadinene	SH	30.43	1739	13.1 ± 13.48	tr	4.61 ± 5.43	nd	nd	nd	RI;MS
[31]	β-curcumene	SH	31.00	1754	3.89 ± 5.01	5.02 ± 11.93	nd	nd	nd	nd	RI;MS
[32]	α-curcumene	SH	31.23	1780	3.03 ± 4.23	tr	5.35 ± 6.82	15.08 ± 4.41	12.09 ± 9.4	23.23 ± 7.41	RI;MS
[33]	nerol	OM	31.84	1796	2.09 ± 1.62	3.05 ± 1.61	2.89 ± 2.57	2.81 ± 2.9	nd	nd	Std
[34]	guaiol	OS	37.56	2101	1.21 ± 1.43	0.7 ± 1	1.19 ± 1.39	0.96 ± 1.32	nd	nd	RI;MS
[35]	rosifoliol	OS	38.32	2149	6.18 ± 7.53	2.04 ± 4.97	5.42 ± 7.8	1.62 ± 1.9	nd	nd	RI;MS

Table S3. Contribution of flower phytochemical compounds on the principal components (Dim.). The values reported are the square coordinates of the variables representing the contribution of the variables to the principal components. A high value indicates a good representation of the variable on the principal component.

	Dim.1	Dim.2	Dim.3	Dim.4	Dim.5
α-pinene	0.46	0.03	0.13	0.00	0.17
β-myrcene	0.35	0.09	0.15	0.08	0.01
α-terpinene	0.00	0.27	0.38	0.07	0.05
limonene	0.41	0.15	0.01	0.04	0.08
1,8-cineole	0.16	0.02	0.09	0.08	0.06
γ-terpinene	0.20	0.13	0.17	0.23	0.00
α-ylangene	0.67	0.13	0.01	0.00	0.00
linalool	0.29	0.00	0.10	0.22	0.16
italicene	0.24	0.08	0.03	0.28	0.05
(E)-α-bergamotene	0.25	0.03	0.03	0.00	0.41
β-caryophillene	0.55	0.11	0.03	0.00	0.00
β-himachalene	0.36	0.26	0.12	0.09	0.06
α-terpineol	0.45	0.23	0.07	0.00	0.00
neryl acetate	0.72	0.09	0.00	0.01	0.00
δ cadinene	0.03	0.44	0.25	0.05	0.00
α-curcumene	0.09	0.21	0.02	0.09	0.01
nerol	0.28	0.11	0.00	0.05	0.11
rosifoliol	0.46	0.03	0.13	0.00	0.17
Proportion of variance	34.35	13.30	8.10	7.44	7.09
Cumulative proportion	32.48	45.78	53.87	61.31	68.40

Table S4. Contribution of leaves phytochemical compounds on the principal components (Dim.). The values reported are the square coordinates of the variables representing the contribution of the variables on the principal components. A high value indicates a good representation of the variable on the principal component.

	Dim.1	Dim.2	Dim.3	Dim.4	Dim.5
α-pinene	0.69	0.00	0.08	0.05	0.01
α-fenchene	0.13	0.25	0.10	0.07	0.00
camphene	0.23	0.07	0.08	0.04	0.00
β-pinene	0.23	0.18	0.23	0.06	0.07
β-myrcene	0.47	0.17	0.01	0.05	0.02
limonene	0.12	0.58	0.09	0.05	0.01
1,8-cineole	0.27	0.06	0.31	0.14	0.03
(Z)-β-ocimene	0.34	0.34	0.01	0.02	0.08
(E)-β-ocimene	0.22	0.38	0.02	0.00	0.07
p-cymene	0.15	0.34	0.00	0.00	0.16
terpinolene	0.48	0.11	0.01	0.06	0.08
α-copaene	0.39	0.01	0.02	0.27	0.02
α-thujone	0.34	0.02	0.02	0.01	0.01
isoitalicene	0.42	0.04	0.27	0.03	0.04
italicene	0.05	0.01	0.03	0.11	0.28
(Z)-α-bergamotene	0.30	0.01	0.14	0.01	0.30
terpinen-4-ol	0.08	0.01	0.02	0.05	0.07
β-caryophillene	0.34	0.05	0.23	0.08	0.08
β-himachalene	0.52	0.01	0.02	0.16	0.00
α-terpineol	0.47	0.08	0.17	0.07	0.04
Sesqui6_UNK	0.26	0.00	0.27	0.07	0.00
neryl acetate	0.63	0.02	0.00	0.23	0.00
δ-cadinene	0.73	0.00	0.03	0.00	0.00
α-curcumene	0.45	0.03	0.09	0.11	0.00
Proportion of variance	34.48	12.09	9.47	7.00	5.73
Cumulative proportion	34.48	46.57	56.04	63.04	68.77

Table S5. Contribution of bark phytochemical compounds on the principal components (Dim.). The values reported are the square coordinates of the variables representing the contribution of the variables to the principal components. A high value indicates a good representation of the variable on the principal component.

	Dim.1	Dim.2	Dim.3	Dim.4	Dim.5
α-pinene	0.75	0.00	0.01	0.00	0.00
camphene	0.26	0.26	0.14	0.11	0.04
β-pinene	0.27	0.21	0.09	0.00	0.03
myrcene	0.19	0.40	0.10	0.00	0.00
limonene	0.52	0.03	0.05	0.10	0.00
γ-terpinene	0.23	0.07	0.20	0.23	0.06
p-cymene	0.02	0.66	0.10	0.01	0.02
α-ylangene	0.25	0.13	0.33	0.00	0.01
α-copaene	0.34	0.20	0.27	0.00	0.01
isoitalicene	0.65	0.00	0.01	0.21	0.01
italicene	0.62	0.00	0.01	0.21	0.00
β-cariophillene	0.20	0.01	0.02	0.11	0.58
neryl-acetate	0.38	0.00	0.01	0.07	0.09
α-curcumene	0.48	0.01	0.01	0.12	0.05
Proportion of variance	36.98	14.98	9.50	8.45	6.40
Cumulative proportion	36.98	51.15	60.65	69.09	75.49
